# New insights on the regulation of the adenine nucleotide pool of erythrocytes in mouse models

**DOI:** 10.1371/journal.pone.0180948

**Published:** 2017-07-26

**Authors:** William G. O’Brien, Han Shawn Ling, Zhaoyang Zhao, Cheng Chi Lee

**Affiliations:** Department of Biochemistry and Molecular Biology, McGovern Medical School, University of Texas Health Science Center, Houston, Texas, United States of America; University of Colorado Denver, UNITED STATES

## Abstract

The observation that induced torpor in non-hibernating mammals could result from an increased AMP concentration in circulation led our investigation to reveal that the added AMP altered oxygen transport of erythrocytes. To further study the effect of AMP in regulation of erythrocyte function and systemic metabolism, we generated mouse models deficient in key erythrocyte enzymes in AMP metabolism. We have previously reported altered erythrocyte adenine nucleotide levels corresponding to altered oxygen saturation in mice deficient in both CD73 and AMPD3. Here we further investigate how these *Ampd3*^*-/-*^*/Cd73*^*-/-*^ mice respond to the administered dose of AMP in comparison with the control models of single enzyme deficiency and wild type. We found that *Ampd3*^*-/-*^*/Cd73*^*-/-*^ mice are more sensitive to AMP-induced hypometabolism than mice with a single enzyme deficiency, which are more sensitive than wild type. A dose-dependent rightward shift of erythrocyte p50 values in response to increasing amounts of extracellular AMP was observed. We provide further evidence for the direct uptake of AMP by erythrocytes that is insensitive to dipyridamole, a blocker for ENT1. The uptake of AMP by the erythrocytes remained linear at the highest concentration tested, 10mM. We also observed competitive inhibition of AMP uptake by ATP and ADP but not by the other nucleotides and metabolites tested. Importantly, our studies suggest that AMP uptake is associated with an erythrocyte ATP release that is partially sensitive to inhibition by TRO19622 and Ca^++^ ion. Taken together, our study suggests a novel mechanism by which erythrocytes recycle and maintain their adenine nucleotide pool through AMP uptake and ATP release.

## Introduction

The principal function of erythrocytes (red blood cells) is delivering oxygen to the body’s tissues, while simultaneously removing carbon dioxide from these tissues. These erythrocyte functions are in part modulated by adenine nucleotides. Both extracellular and intracellular levels of adenine nucleotides are tightly regulated by several well studied enzymes. Extracellularly, there is an ATPase (CD39) that converts ATP to ADP and then to AMP [1]. An extracellular ectonucleotidase (CD73) further dephosphorylates AMP to adenosine [2]. Adenosine is either taken up through nucleoside transporters such as ENT1/2 or quickly catabolized by adenosine deaminase (ADA). Intracellular adenosine can be phosphorylated by adenosine kinase, forming AMP. AMP can be further phosphorylated (with ATP as the phosphate donor) to produce ADP by adenylate kinase [3]. Another fate of intracellular AMP is deamination to inosine monophosphate (IMP) by AMP deaminase (AMPD). There are three tissue specific isoforms of AMPD; AMPD1, 2, and 3 are muscle, liver and erythrocyte-specific isoforms, respectively. In nucleated cells, IMP can be converted back to AMP by adenylosuccinate synthase and lyase. Thus, nucleotide degradation products can be salvaged to replenish the adenylate pool. Unlike nucleated cells, erythrocytes do not carry out de novo purine biosynthesis nor do they have a salvage pathway for IMP [4]. Erythrocyte AMP is mainly irreversibly catabolized by AMPD3, while a stable adenylate equilibrium is controlled by adenylate kinase. Having a similar regulatory environment for extracellular adenine nucleotides to other cell types, erythrocytes appear to have a unique mechanism to take up extracellular AMP efficiently [5][6].

Our earlier investigations identified AMP as a biomolecule that could induce hypometabolism, termed AMP induced hypometabolism (AIHM) [7], a state in which the metabolic rate of the animal is reduced to about 10% of the resting level. Our studies suggested that the erythrocyte is a key target of AIHM [5]. We demonstrated the length of AIHM is not dose dependent. Induction of AIHM is specific to adenine nucleotides but not other nucleotides [5]. It had been proposed by others that AIHM was an adenosine receptor mediated mechanism since it was observed that AMP administration led to rapid slowing of the heart rate [8], suggesting adenosine receptor (A1a) mediated bradycardia was the underlying mechanism for AIHM. However, we observed that wild type, *A1a*^*-/-*^, *A2a*^*-/-*^, *A2b*^*-/-*^, and *A3a*^*-/-*^ mice respond to AIHM in a similar fashion [5], demonstrating that the disruption of adenosine receptor function did not alter AIHM. Interestingly, the rapid decline in heart rate in response to AMP seen in wild type mice was absent in *A1a*^*-/-*^ mice, suggesting bradycardia is not the cause but a result of AIHM [9]. Further, we observed that *Cd73*^*-/-*^ mice were more sensitive to a lower dose of AMP than wild type mice, suggesting that generation of adenosine via dephosphorylation of AMP by CD73 actually impeded the process of AIHM [5].

To further establish the role of the erythrocyte in AIHM, we investigated the effect of altering AMP degradation within erythrocytes. There are two pathways for AMP catabolism: either through its dephosphorylation to adenosine, and then to inosine via ADA, or through its deamination to inosine monophosphate (IMP). Given that the K_m_ of ADA for adenosine is two orders of magnitude larger than that of adenosine kinase [10], we reasoned that the majority of AMP catabolism must be via its deamination to IMP. Since erythrocytes do not have a salvage pathway for converting IMP back to AMP, the catabolism of AMP by AMP deaminase 3 (AMPD3) would be irreversible. We created a strain of mouse deficient in AMPD3, the *AMPD3*^*-/-*^ model [11]. Unexpectedly, we observed that erythrocyte AMP levels did not change significantly; rather, both ATP and ADP levels increased by about 3 fold, indicating that ratios among the adenine nucleotides are tightly regulated in erythrocytes. Additionally, we observed that *Ampd3*^*-/-*^ mice, like the *Cd73*^*-/-*^ mice, were more sensitive to AIHM than wild type mice. These studies with the single deficiency models supported that both CD73 and AMPD3 modulate AIHM. They further supported the central role that erythrocytes play in mediating the mechanism of AIHM. In order to further our understanding of the role of adenine nucleotides in erythrocyte function, we created a mouse model that was deficient in both AMPD3 and CD73, the key intracellular and extracellular AMP catabolic enzymes respectively. We have recently reported an investigation into the physiological importance of CD73 and AMPD3 to the physiological function of an erythrocyte, particularly oxygen transport and overall metabolism [12]. We showed that increased adenine nucleotide levels, ATP in particular, correlate with increases in erythrocyte p50 values. Alteration of AMP catabolism in these models also significantly affects systemic metabolism.

In this study, we have initiated an investigation into how CD73 and AMPD3 deficiencies impact the response to AIHM and how erythrocyte oxygen transport is affected when exposed to a large dose of AMP in AIHM. These studies further led us to investigate how the adenylate pool in erythrocytes is regulated in response to a large dose of AMP. Our findings suggest that the erythrocyte has a previously poorly understood capability of regulating its own adenylate pool.

## Materials & methods

### Materials

Radiolabelled nucleotides were acquired from Moravek Biochemicals. Solutions for the Hemox-Analyzer were acquired from TCS Scientific Corporation. Phosphate Buffered Saline (BP399-1, 10X PBS solution diluted to 1X prior to use with final concentrations of 11.9 mM Phosphates, 137 mM Sodium Chloride, and 2.7 mM Potassium Chloride) was acquired from Fisher Scientific. TRO19622 was acquired from Tocris Bioscience (Bristol, United Kingdom). All other chemicals and reagents were purchased from Sigma.

### Mouse housing/husbandry/protocols

All animal studies were carried out in the institutional animal facility by trained personnel under protocols HSC-AWC-13-012 and HSC-AWC-12-079 approved by the Animal Welfare Committee (AWC), the institutional animal care and use committee (IACUC) at UTHSC-Houston. Wild type mice were ordered from Jackson Laboratory (Bar Harbor, Maine). The Cd73^-/-^ mice were obtained from Dr. L. Thompson [13]. The Ampd3^-/-^ mice were generated as previously reported [11]. The Cd73^-/-^/Ampd3^-/-^ mice were generated by breeding the Cd73^-/-^ mice with the Ampd3^-/-^ mice followed by backcrossing the F1 mice. Mice were maintained in the animal facilities with a 12h:12h light:dark cycle at an ambient temperature of ~23°C. Mice had access to standard rodent chow and water ad libitum and their cages were changed twice a week.

### Metabolic chamber experiments

A Comprehensive Lab Animal Monitoring System (CLAMS, Columbus Instruments, Columbus, OH) was used to measure animal metabolic rate as previously described [5]. Briefly, each chamber contained an individual mouse with free access to food and water. The O_2_ consumption and CO_2_ production of each mouse was monitored and recorded with the OxyMax System from Columbus Instruments. Each chamber has the option of wheel addition for running. The chamber was maintained at normal husbandry conditions at 23°C under a 12h:12h LD cycle.

### Erythrocyte isolation

Whole blood was collected from a tail clip and allowed to drip into a heparinized Eppendorf tube. The whole blood was then centrifuged at 4°C at 3000 rpm for one minute. The plasma was then removed and the erythrocytes were washed 3X by resuspension in a 10X volume of ice-cold phosphate buffered saline (PBS) and centrifugation was repeated. After a final wash, the resulting erythrocyte pellet was resuspended with a small volume of PBS to about the original whole blood volume. The resulting suspension was counted on a hemacytometer for the number of erythrocytes after two serial 1:100 dilutions. Based on the cell count, the isolated erythrocytes were then diluted with PBS to obtain about 10^6^ cells/μL. For oxygen saturation experiments, TLC experiments, AMP uptake experiments, and ATP release experiments, 10 μL of 10^6^ cells/μL was used; for adenine nucleotide extraction experiments, 75 μL of 10^6^ cells/μL was used. Erythrocytes from mice of all the genotypes were prepared in a similar manner. Isolated erythrocytes were CD73^-/-^ unless otherwise noted to ensure AMP did not degrade to Ado.

### Blood oxygen saturation experiments

All blood oxygen saturation experiments were measured with a Hemox Analyzer from TCS Scientific Corporation as described by Guarnone *et al*. [14]. Solutions for the Hemox Analyzer were also acquired from TCS Scientific Corporation. Briefly, 10 μL of 10^6^/μL of erythrocytes were incubated in 4 mL of Hemox buffer, 10 μL anti-foaming agent (AFA-25, TCS Scientific Corporation) and 0.1% BSA and equilibrated at 37°C. In experiments where lysates were used, 10 μL of 10^6^ cells/μL of erythrocytes was treated by freeze-thawing twice to generate the lysate. Once the sample in the Hemox Analyzer had equilibrated to a pO_2_ of 150 mmHg and a temperature >36.7°C, measurement of oxygen saturation was initiated by perfusing the sample with Nitrogen gas to deoxygenate the hemoglobin. The analysis of p50 was complete once the pO_2_ was below 2 mm Hg.

### Adenine nucleotide measurements

Adenine nucleotide measurements were carried out as described by Stocchi *et al*. [15]. Briefly, 2 volumes of 7.5% perchloric acid were added to isolated erythrocytes, mixed by vortexing, and then centrifuged at 4°C for 10 min at 13,000 x g. The supernatant was neutralized with an equal volume of 0.75 M K_2_CO_3_. After neutralization, the reaction mixture was centrifuged at 4°C for 10 min at 13,000 x g and the resulting supernatant was injected onto a pre-equilibrated HPLC C-18 column. The HPLC elution gradient was as described by Bhatt *et al*. [16]. Buffer A was 30 mM KH_2_PO_4_ + 0.8 mM TBAP (tetrabutylammonium phosphate) pH 5.45. Buffer B was 50% (v/v) acetonitrile in 30 mM KH_2_PO_4_ + 0.8 mM TBAP, pH 7.0. After each run, a 20 min flush with Buffer A was carried out to re-equilibrate the column.

### Injection of AMP

Mice were fasted overnight and an intraperitoneal injection with the desired dose of 5’-AMP in sterile PBS was given early in the morning to induce the 5’-AMP Induced Deep Hypometabolic (AIHM) state. The metabolic chamber was maintained at ~15°C during these experiments in order to facilitate the induction and maintenance of the AIHM state. The animals were monitored via the CLAMS computer readout and were removed from the metabolic chamber and placed in their room temperature cage upon arousal from the AIHM state. We defined the AIHM state as occurring when an animal’s VO_2_ falls below 1200 mL/kg/h, since the mice tend to enter the arousal state when their VO2 rise above 1200 mL/kg/h. We have observed that most mice begin arousal and rapid re-warming not long after reaching a VO_2_ of 1500 mL/kg/h.

### Incubation of erythrocytes with adenosine/AMP/other nucleotides

Adenosine (Ado) was dissolved in DMSO to make a stock of 200 mM, which was then diluted to the appropriate concentration with PBS. Solutions of the other nucleotides were prepared similarly but with PBS as the solvent. After addition of nucleotide, the erythrocyte suspension was shaken at 750 rpm in a thermomixer at 37°C. At the desired time, the erythrocyte suspension was removed and processed according to the experimental protocol.

### TLC of radiolabelled ADP/AMP

Isolated erythrocytes (13 μL cells) were incubated at 37°C with 2 μL of stock [^14^C]-AMP or [^14^C]-ADP for the desired time and then centrifuged at 4°C for 1 min at 3000 x g to separate cells from supernatant. The supernatant was removed and saved for future analysis. The cell pellet was washed twice with 1 ml of cold PBS, and the final cell pellet was lysed by mixing rapidly with 15 μL of 5% TCA. The reaction mixture was centrifuged and the final supernatant saved for TLC analysis. Both the initial supernatant and the final supernatant were then spotted (1 μL at a time, with a hairdryer used to speed up drying) on plastic TLC plates coated with silica gel 60 and F254 fluor (Merck). The TLC plates were developed with butanol / methanol / water / ammonium hydroxide (60:20:20:1). The TLC plate was then air dried and the radioactive bands visualized by exposure to X-ray film at -80°C.

### Uptake of AMP using ^14^C-AMP

Isolated erythrocytes were incubated at 37°C with various concentrations of AMP at a constant specific activity. After 45 min incubation at 37°C, all reactions were centrifuged to separate cells from supernatant. The supernatant was removed and kept for further analysis. The cell fraction was washed twice with 1 ml cold PBS, and the final cell pellet was lysed by mixing with 15 μL of 5% TCA. The amount of radioactivity in the supernatant and cell fractions was determined by adding 10 mL of ScentiSafe Econo 2 scintillation cocktail (Fisher) and vials were counted for 5 min each using a scintillation detector [Pharmacia Rackbeta #1209–015]. In experiments where an inhibitor was used, the inhibitor was pre-incubated with the erythrocytes for 5 min prior to addition of AMP. In competition experiments with other nucleotides/metabolites, each competitor was added at the same time as AMP.

### ATP release measurements

ATP released by erythrocytes was measured using a Perkin Elmer ATP luciferase kit following the manufacturer’s instructions. Initial experiments were recorded using a 96-well plate luminometer [Envision Multilabel Reader (Perkin Elmer) #2104–0010] in real time. In the experiments with inhibitors, the erythrocytes were preincubated with inhibitor for 5 min before addition of AMP.

### Statistics

All values plotted are averages +/- SEM, with the significance of any differences evaluated by either t-test or ANOVA, as appropriate for the data set.

## Results

### Analysis of AIHM and torpor response in mice with deficiencies in both CD73 and AMPD3

**Fig 1:**
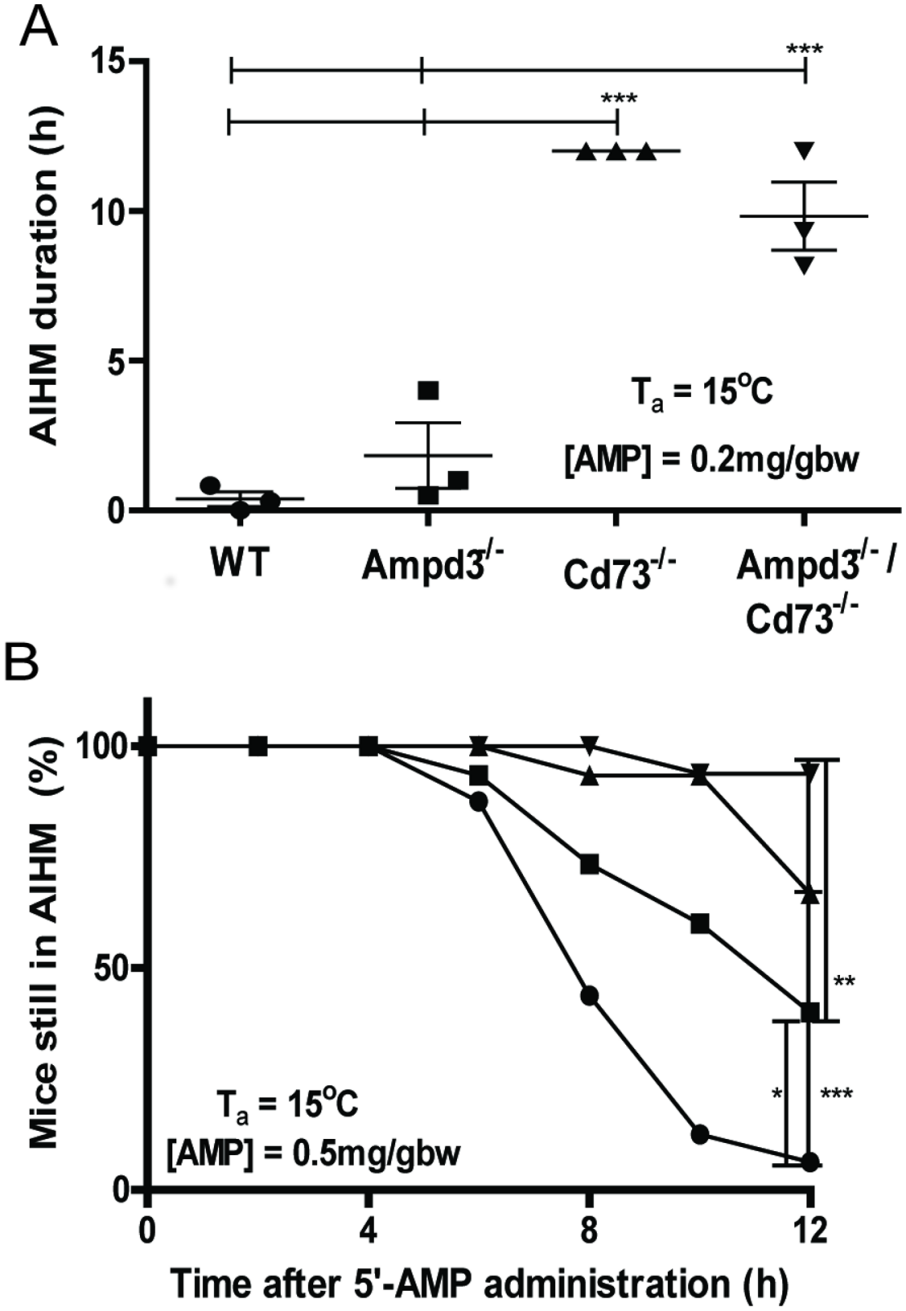
AIHM responses among four genotypes of mice. (A). AIHM duration varies among four genotypes of mice. Mice from each genotype (N = 3) were injected with a sub-optimal dose of AMP (0.2 mg/gbw) and placed at an ambient temperature of 15°C. The amount of time in AIHM was calculated from the time of injection to when they increased their VO_2_ to 1200 mL/kg/h or, in the case of the wild type (WT) mice, until their VO_2_ started to increase. (B). Graphical representation of the percentage of mice remaining in AIHM relative to total injected animals as a function of time. Mice from each genotype (wild type (N = 16), *Ampd3*^*-/-*^ (N = 15), *Cd73*^*-/-*^ (N = 15), and *Ampd3*^*-/-*^*/Cd73*^*-/-*^ (N = 16)) were injected with the optimal dose of AMP (0.5 mg/gbw) and placed at an ambient temperature of 15°C. The length of time each mouse stayed in AIHM was calculated from injection to when their VO_2_ rose above 1200 mL/kg/h. Sampling of animals in AIHM were taken every 2 h. P values = * < .05, ** < .005, and *** < .0001.

To examine how the *Ampd3*^*-/-*^*/Cd73*^*-/-*^ mice respond to AIHM compared to wild type or mice deficient in either AMPD3 or CD73, we compared the length of time mice remained in AIHM or induced torpor in response to a sub-optimal dose of AMP (0.2 mg/gw) to see if the *Ampd3*^*-/-*^*/Cd73*^*-/-*^ mice were more sensitive to AMP [5]. Throughout this experiment, the mice were kept in a Comprehensive Lab Animal Monitoring System (CLAMS) at an ambient temperature (T_a_) of 15°C and the VO_2_ of individual animals was recorded. For the analysis of this experiment, we considered an animal to be in a torpor state when its VO_2_ dropped below 1200 mL/kg/h. We previously have shown that mice deficient in either CD73 or AMPD3 stayed in AIHM longer than wild type mice when given a lower dose of AMP [5][11]. Here, we observed that mice deficient in both CD73 and AMPD3 remained in AIHM for an average length of 9 h, while the majority of wild type mice failed to enter the AIHM state, with VO_2_ levels staying above 1200 ml/kg/h after injection of the sub-optimal dose of AMP (Fig 1A). The VO_2_ of the majority of the *Ampd3*^*-/-*^ mice dropped below 1200 mL/kg/h for an average period of 2 h. The *Cd73*^*-/-*^ mice showed greater responses than the *Ampd3*^*-/-*^ mice, typically staying in AIHM for an average of 6 h.

Next, we compared the length of AIHM among the different genotypes upon receiving the optimal dosage of AMP (0.5 mg/gw). At this dosage, all the mice in these studies entered deep AIHM at 2 h following administration of AMP. The cohort of wild type mice had the shortest average length of AIHM (Fig 1B, Fig iA in S1 File). Over 90% of wild type mice had already aroused from AIHM at 10 h following AMP administration. In contrast, at 10 h, over 60% of *Ampd3*^*-/-*^ mice and about 95% of *Cd73*^*-/-*^ and *Ampd3*^*-/-*^*/Cd73*^*-/-*^ mice were still in AIHM (Fig 1B, and Fig iB-D in S1 File). The difference between *Cd73*^*-/-*^ and *Ampd3*^*-/-*^*/Cd73*^*-/-*^ mice became apparent 12 h after AMP administration when about 65% of *Cd73*^*-/-*^ mice remained in AIHM but no change was observed for *Ampd3*^*-/-*^*/Cd73*^*-/-*^ mice. After 14 h, when the experiment was terminated as required by our AWC protocol, the number of *Ampd3*^*-/-*^*/Cd73*^*-/-*^ mice that remained in the AIHM state had not changed. Together, these data demonstrate that CD73 and AMPD3 regulate the potency and length of AIHM.

### Manipulation of extracellular AMP modulates erythrocyte p50

**Fig 2:**
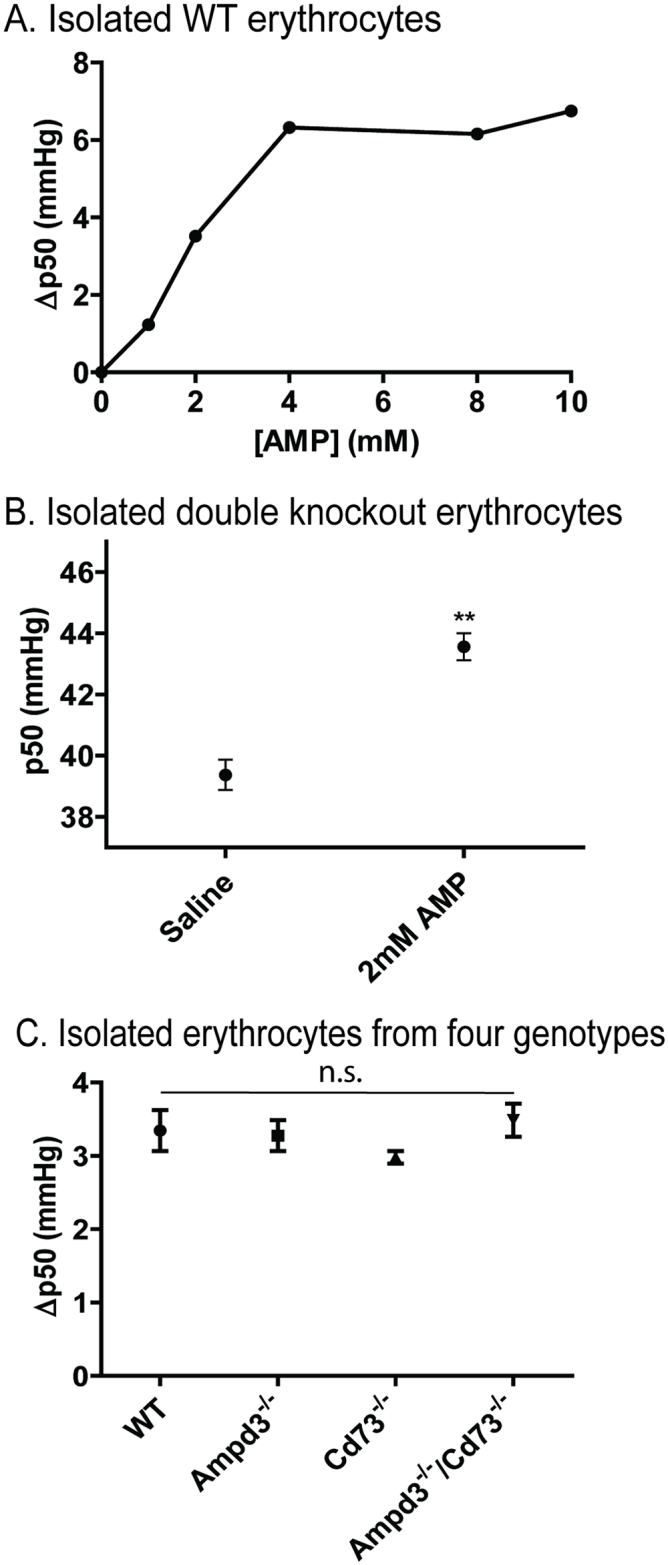
Effect of AMP on the p50 of intact erythrocytes. (A) Shift of WT erythrocytes’ p50 in response to extracellular AMP concentration; (B) Average AMPD3^-/-^/CD73^-/-^ erythrocyte p50 values in PBS and 2 mM AMP (N = 3). (C) Shift of erythrocytes’ p50 after 2 mM AMP incubation in the four genotypes (N = 3).

In our recently published study, we observed that both AMPD3 deficient and CD73 and AMPD3 double deficient erythrocytes displayed increased p50 values as a result of an increase in physiological intracellular adenine nucleotide levels [12]. We reasoned that the observed increased response to AMP in inducing the AIHM state could be associated with direct or indirect effects of AMP on the p50 value of erythrocytes. Here, we measured the response of erythrocytes to extracellular AMP. We observed an increase in p50 corresponding to increases in extracellular AMP levels (Fig 2A). Interestingly, the ability of AMP to further shift p50 diminished above 4 mM, suggesting AMP is regulating the oxygen carrying capacity of erythrocytes by a saturable mechanism. Due to the perceived saturable component, but still wanting to see a significant shift in the p50 curve, we tested 2 mM extracellular AMP on isolated mouse *Ampd3*^*-/-*^*/Cd73*^*-/-*^ erythrocytes (Fig 2B); we observed an increase in p50 of about 4 mm Hg and a rightward shift in the oxygen saturation curve suggesting that extracellular AMP is lowering the erythrocytes’ affinity for oxygen. When p50 changes in response to 2 mM extracellular AMP were measured in isolated erythrocytes of all four genotypes, the p50 shifts were comparable (Fig 2C). *Ampd3*^*-/-*^*/Cd73*^*-/-*^ erythrocytes were used to exclude the possibility of an indirect effect of AMP via dephosphorylation to adenosine. In addition, we carried out similar experiments at the same concentration of adenosine (2 mM) with isolated *Ampd3*^*-/-*^*/Cd73*^*-/-*^ erythrocytes. Compared to the vehicle solution, the addition of adenosine did not alter the erythrocytes’ p50 significantly (Fig ii in S1 File). This result further excluded the possibility that this shift in p50 was due to adenosine.

### Direct uptake of AMP by erythrocytes

We showed that erythrocytes could take up AMP [5], but we could not rule out the possibility that the p50 shift induced by extracellular 5’-AMP could be partially mediated by a previously unrecognized AMP-receptor mediated mechanism. We reasoned that a receptor based mechanism depends on the integrity of the cell. Therefore, if extracellular AMP depends on a receptor-mediated mechanism to induce the p50 shift, then this response should be abolished when erythrocytes were lysed. We observed a linear p50 shift that correlated with AMP concentration in the erythrocyte lysate that has a slope similar to the intact erythrocyte (Fig iii in S1 File), suggesting that the regulation by extracellular AMP of erythrocyte’s p50 is not dependent on the integrity of the erythrocyte cell membrane. Thus, a receptor mediated mechanism is unlikely.

**Fig 3:**
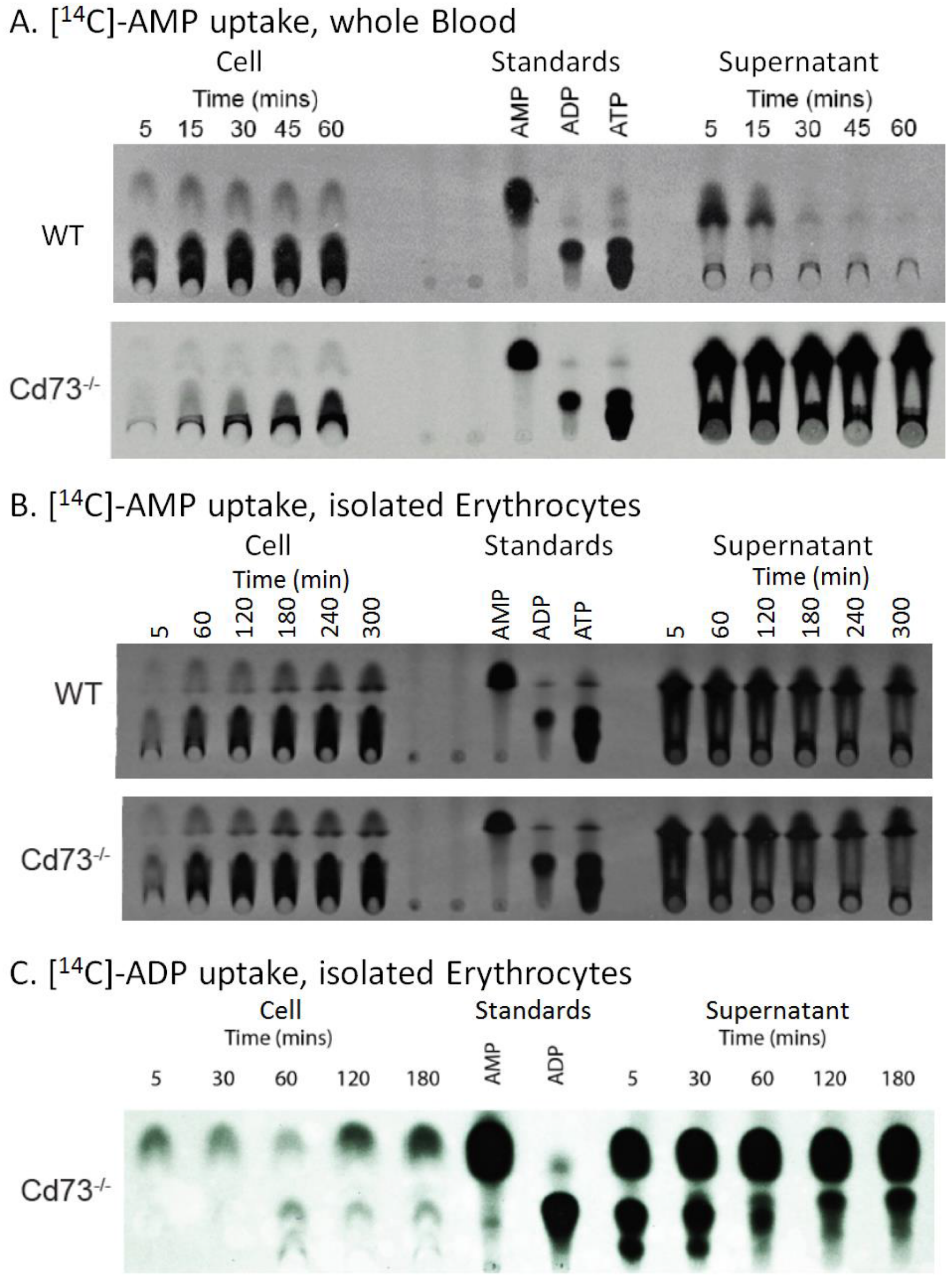
Stability and uptake of extracellular AMP and ADP. (A) Stability and uptake of extracellular AMP by erythrocytes in whole blood. Whole blood incubation of [^14^C] AMP from wild type (WT) and *Cd73*^*-/-*^ mice. (B) Isolated erythrocyte incubation of [^14^C] AMP from WT and *Cd73*^*-/-*^ mice. Note: x-ray film exposure time was selected to favor signals in the supernatant. (C) Fate of ADP incubated with isolated erythrocytes. TLC analysis showing the fate of ADP in cell and supernatant fractions when isolated erythrocytes were incubated with [^14^C] ADP.

Although uptake of AMP by erythrocytes has been independently observed [6][5], its characteristics and kinetics remain to be investigated. Erythrocytes have equilibrative nucleoside transporters (ENTs) that readily uptake adenosine, a product of AMP dephosphorylation. A question was raised on whether AMP was taken up directly or indirectly as a result of rephosphorylated adenosine. To address this question, we first verified that the dephosphorylation of AMP to adenosine by CD73 is truly absent in *Cd73*^*-/-*^ mouse blood, using wild type mouse blood as a control. Cell lysates and supernatants from a time-course of incubation of [^14^C]-AMP with whole blood from the two genotypes were profiled by thin-layer chromatography (TLC) (Fig 3A). Interestingly, in cell fractions of both genotypes, the TLC plates displayed detectable levels of [^14^C]-AMP intracellularly, even at 5 min incubation, suggesting that AMP had already entered the erythrocytes. In addition, some intracellular ADP was formed from [^14^C]-AMP through the adenylate equilibrium, similar to our earlier observation [5]. The TLC analysis further revealed that the majority of extracellular [^14^C]-AMP was already catabolized in wild type whole blood supernatant after a 15 min incubation at 37°C. By contrast, the majority of extracellular [^14^C]-AMP remained intact in the supernatant of *Cd73*^*-/-*^ whole blood even after 1 h incubation at 37°C. These findings are consistent with CD73 being the major extracellular catabolic enzyme for AMP [17] and give confidence that studies using tissues deficient in CD73 would significantly eliminate dephosphorylation of extracellular AMP into adenosine.

Next, we investigated whether isolated erythrocytes dephosphorylate extracellular AMP into adenosine, addressing the question of whether CD73 or other AMP catabolic enzymes are directly associated with erythrocytes. Isolated and washed erythrocytes were incubated with [^14^C]-AMP over a 5 h time course at 37°C. Cell and supernatant fractions were separated and analyzed by TLC (Fig 3B). Isolated erythrocytes from either wild type and *Cd73*^*-/-*^ mice did not catabolize extracellular [^14^C]-AMP significantly even after 5 h at 37°C. In addition, the uptake of [^14^C]-AMP into the erythrocytes was comparable between the two genotypes. These studies demonstrated that uptake of [^14^C]-AMP by erythrocytes is direct and that CD73 is not associated directly with erythrocytes.

We further investigated whether erythrocytes could also directly uptake other adenine nucleotides. Since it has been demonstrated that CD39/ATPase converts ATP into ADP and then into AMP extracellularly, we investigated whether [^14^C]-ADP is taken up directly. Isolated *CD73*^*-/-*^ erythrocytes were incubated with [^14^C]-ADP over a 3 h time course. Cell and supernatant fractions were then separated at specific time points and analyzed by TLC (Fig 3C). We observed that significant levels of [^14^C]-ADP in the supernatant fraction had already been dephosphorylated into [^14^C]-AMP after 5 min of incubation with isolated erythrocytes at 37°C. In the cell fraction, within the first 5 min we observed [^14^C]-AMP but no apparent levels of [^14^C]-ADP. No significant level of [^14^C]-ADP was detected in the cells even after 30 min incubation. By this time, the majority of the supernatant [^14^C]-ADP had already been dephosphorylated to [^14^C]-AMP. After 1 h, both [^14^C]-AMP and [^14^C]-ADP were detected inside the erythrocytes but the increase in [^14^C]-ADP coincided with decreases in intracellular [^14^C]-AMP levels suggesting that the rise in [^14^C]-ADP could be a result of the erythrocytes’ adenylate equilibrium rebalancing the cellular adenine nucleotide ratio rather than via ADP uptake by the erythrocytes. Together, these studies suggest that the outer membrane of erythrocytes contained CD39/ATPase but not CD73 or another 5’-nucleotidase and that erythrocytes readily uptake AMP but not ADP.

### Further characterizations of AMP uptake by erythrocytes

**Fig 4:**
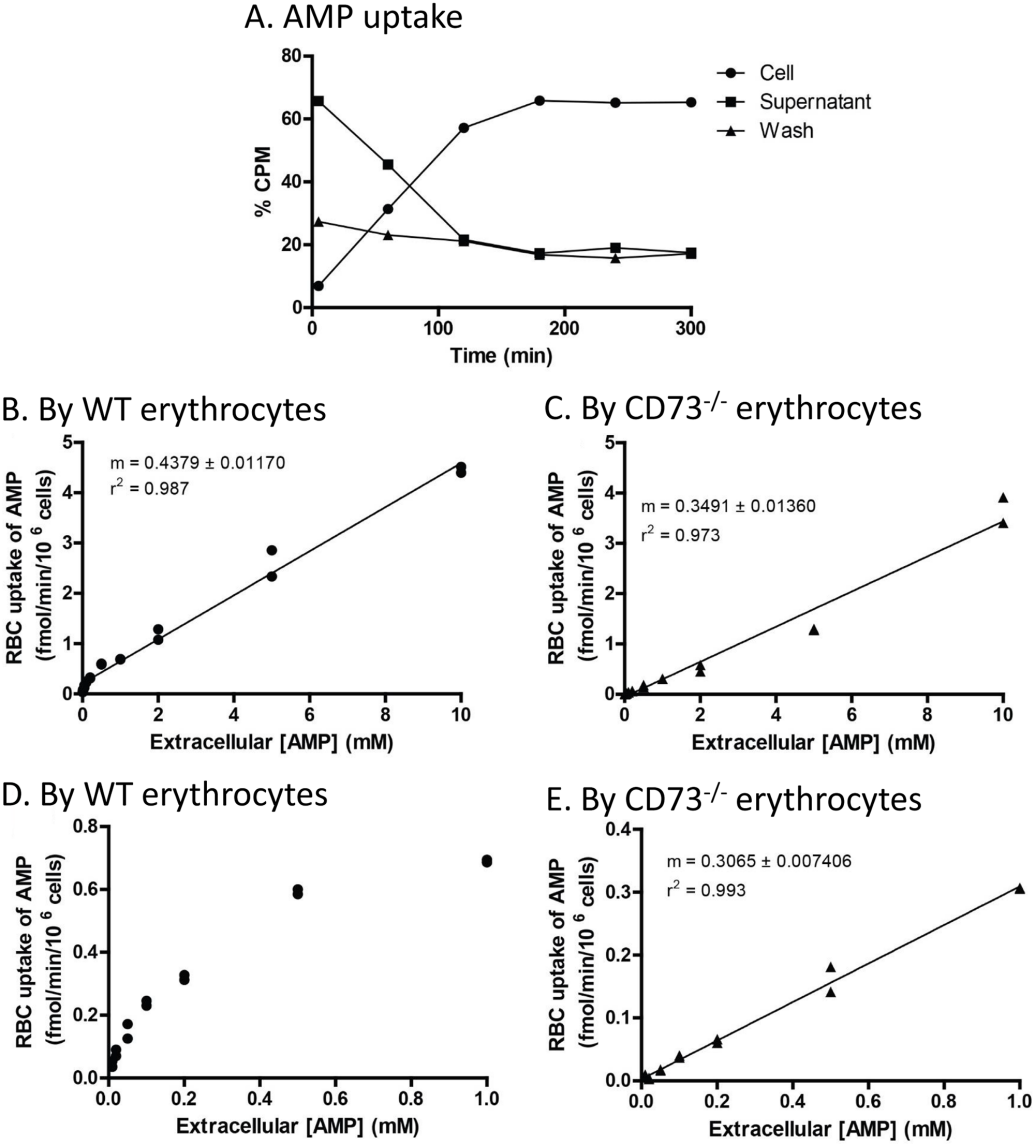
Concentration dependent uptake of AMP by erythrocytes. (A) A typical assessment of AMP uptake by erythrocytes. [^14^C]-AMP signal as a percentage of total added radioactivity in the supernatant, cells, and wash fractions over a time course. Uptake of AMP by wild type (B) and *Cd73*^*-/-*^ (C) erythrocytes up to 10 mM extracellular AMP were measured. The difference in AMP uptake by the two genotypes at low extracellular AMP concentrations is exhibited by plots of uptake in the range of 0–1 mM extracellular AMP concentrations in wild type (D) and *Cd73*^*-/-*^ (E) erythrocytes.

To better understand the uptake of AMP by erythrocytes, we investigated the kinetics of this uptake process. We reasoned that if this uptake was enzymatically mediated, we should be able to calculate the K_m_ and V_max_ of the corresponding protein. Radiolabelled [^14^C]-AMP was incubated with isolated wild type mouse erythrocytes at 37°C. At specific time points, aliquots of the reaction mixture were separated by centrifugation into cell and supernatant fractions. The percentages of the radioactivity from the three fractions were plotted as a function of time of incubation (Fig 4A). We observed that the percentage of radioactivity in the wash fraction was constant over the entire time course. The supernatant fractions had the highest percentage of radioactivity at the beginning of the time course, but the levels declined over time. Corresponding to this decline, the radioactivity in the cell fraction, which was lowest at the beginning of the time course, now increased over time. We observed a reciprocal inversion of radioactivity between cell and supernatant fractions, which was linear during the initial 2 h of the incubation.

Based on conditions established in the experiment above, the amount of AMP uptake was measured as a function of extracellular AMP concentration using a 45 min incubation period at 37°C. We observed that erythrocytes’ uptake of AMP was linearly related (r^2^ = 0.97) to the extracellular concentration of AMP up to 10 mM for isolated wild type (Fig 4B) and *Cd73*^*-/-*^ erythrocytes (Fig 4C). There was no major difference between wild type and *Cd73*^*-/-*^ erythrocytes at extracellular AMP concentrations above 1 mM. However, at concentrations below 1 mM, we observed a “saturable” component in wild type but not *CD73*^*-/-*^ erythrocytes (Fig 4D and 4E). We reasoned that wild type samples have trace amounts of CD73, likely from the low level of white blood cells that were not completely removed when the erythrocytes were isolated. CD73 dephosphorylates extracellular AMP to adenosine, which can then enter the erythrocytes via the ENTs. This small adenosine uptake due to the trace amount of CD73 “contamination” in the wild type isolated erythrocytes is presumably what is responsible for the “saturable” component observed. Above 0.1 mM extracellular concentration, AMP uptake was linear, thus K_m_ and V_max_ values could not be determined. Together, these findings suggest that erythrocytes have a very large relative capacity to uptake extracellular AMP, presumably through a pore or channel.

**Fig 5:**
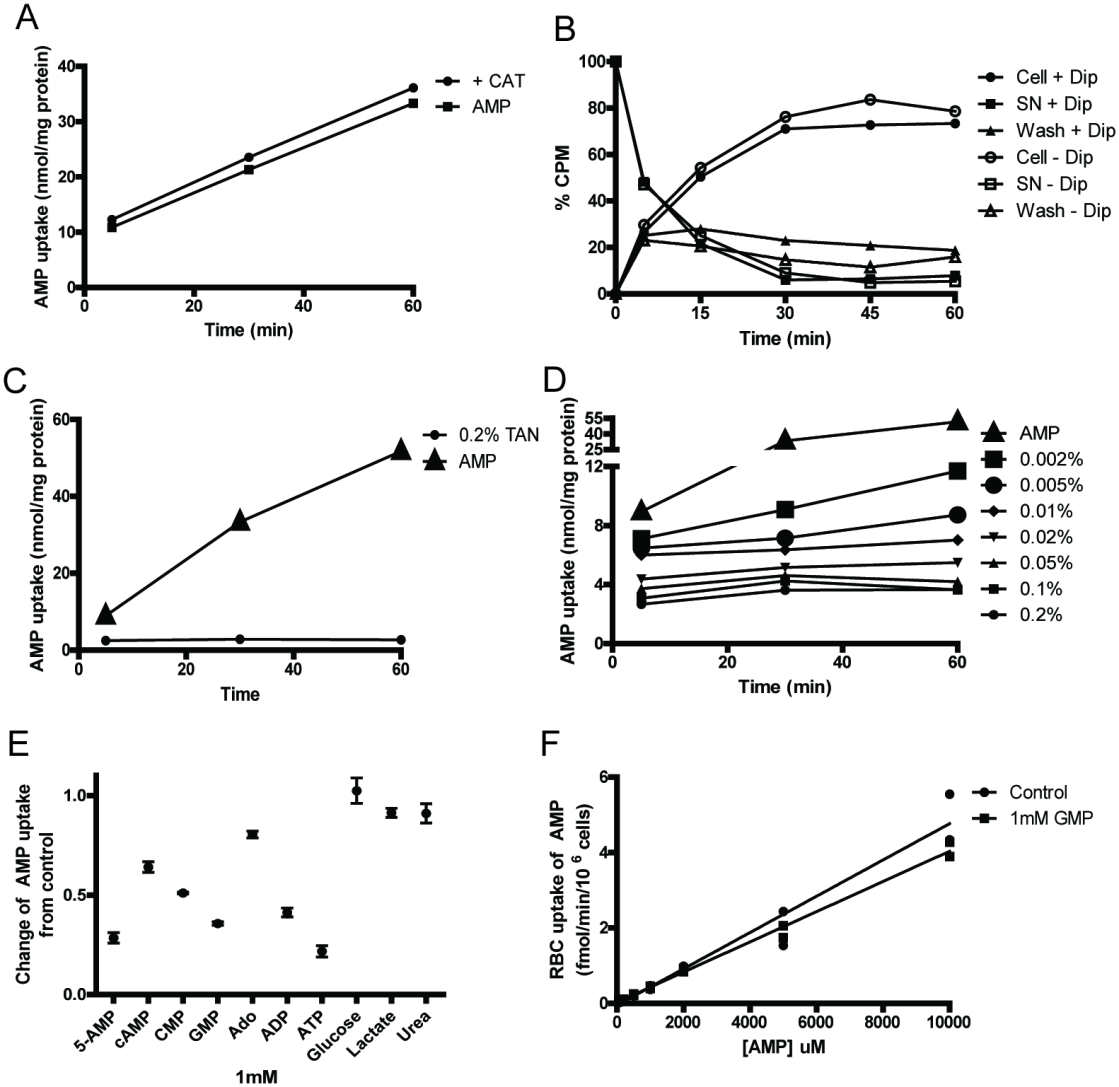
Evaluation of potential inhibitors of AMP uptake by erythrocytes. (A) CAT effect on erythrocyte AMP uptake. (B) Dipyridamole (Dip) effect on erythrocyte AMP uptake. (C) The inhibition of erythrocyte AMP uptake by Tannic Acid (TAN). (D) Titration of TAN concentration in percentages against erythrocyte AMP uptake. (E). AMP uptake inhibition assay to evaluate possible competitive nucleotide/metabolite inhibitors for AMP uptake. (F) Examining GMP inhibition of AMP uptake as a function of increasing concentrations of extracellular AMP.

We surmised that this linear uptake could be mediated by membrane pores or specific channels. However, there is(are) no known channel(s) for transporting AMP across the erythrocyte membrane. There are known adenine nucleotide transporters, ATP-ADP translocases (ANT), which are members of the solute carrier family 25 (slc25) proteins in the mitochondria (SLC25a 4–6 and 31). The mitochondrial ANT’s are sensitive to carboxyatractyloside (CAT) inhibition [18]. Therefore, we carried out an uptake study to determine if CAT had an impact on AMP uptake by erythrocytes. We observed that the erythrocytes’ uptake of AMP was not significantly affected by the addition of CAT (Fig 5A). ENT1, known to mediate the uptake of adenosine, is sensitive to inhibition by dipyridamole. To examine whether ENT1 expressed within the erythrocyte membrane could possibly also mediate AMP uptake, we measured AMP uptake of erythrocytes in the presence of 10 μM dipyridamole. We found that AMP uptake was not significantly affected by the addition of dipyridamole (Fig 5B). Next, we investigated whether the chloride channel inhibitor Tannic Acid (TAN), which has been shown to be a highly effective inhibitor of the adenine nucleotide transporter (SLC25a) family and Ca^2+^ and Cl^-^ ion channels [19], had an effect on AMP uptake. We observed a strong inhibitory effect on erythrocytes’ uptake of radiolabeled [^14^C]-AMP by 0.2% of TAN, a commonly used dose (Fig 5C) [20][21]. Next, we carried out a titration of the effective dose of TAN for inhibition of AMP uptake (Fig 5D). We observed that the amount of AMP uptake by the erythrocytes was significantly reduced when the concentration of TAN decreased to below 0.01%. We concluded that AMP uptake was sensitive to TAN interaction with the erythrocytes. Together, these studies suggest that uptake of AMP by erythrocytes is insensitive to CAT and dipyridamole, but is sensitive to TAN inhibition.

Next, we investigated whether erythrocytes’ AMP uptake is competitively inhibited by cellular metabolites. We examined various common cellular metabolites including ATP, ADP, adenosine, cAMP, CMP, GMP, glucose, lactate, and urea. The uptakes of [^14^C]-AMP by wild type erythrocytes with and without 1 mM of the competing metabolite were compared (Fig 5E). We observed that glucose, urea, lactate, and adenosine had minimal effect on AMP uptake by erythrocytes. While all nucleotides tested show some level of inhibition of or competition with [^14^C]-AMP uptake, cAMP is the least effective followed by CMP. The inhibitory effect of ATP was as strong as unlabeled AMP, while ADP was only slightly less effective. Interestingly, GMP appeared comparable to ADP in inhibiting uptake or in competing with AMP for uptake by erythrocytes. We further characterized this putative inhibition by GMP of AMP uptake by erythrocytes by comparing the effect of increasing extracellular AMP in the presence and absence of 1 mM GMP (Fig 5F). We observed that the rate of AMP uptake was driven by the extracellular concentration and was not inhibited by GMP. Together these studies suggest that the putative channel/pore for AMP uptake by erythrocytes has some level of specificity for adenine nucleotides.

Using HPLC, we examined intracellular adenine nucleotide levels in isolated erythrocytes that were incubated with varying concentrations of extracellular AMP up to 20 mM, at 37°C for 15 min. We observed that AMP uptake led to a change in intracellular adenine nucleotide levels in erythrocytes (Figs iv-vi in S1 File). The erythrocytes from all the genotypes in these studies showed similar correlative trends, though the levels of the untreated erythrocytes’ intracellular adenine nucleotides were 3 times higher in the *AMPD3*^*-/-*^ genotypes. This observation is consistent with the fact that AMPD3 is the gateway enzyme to the main catabolic pathways for the adenine nucleotide pool in erythrocytes. Similar to the AMP uptake experiments using [^14^C]-AMP, the HPLC approach also revealed a linear uptake of AMP into the cell that is driven by the concentration of extracellular AMP (Fig iv in S1 File). We observed that the drop in the intracellular ATP:AMP ratio is inversely exponential (r^2^ = 0.991) to extracellular AMP concentrations (Fig vii in S1 File). Our analysis revealed a small increase in intracellular AMP resulting in a large drop of intracellular ATP levels, especially when extracellular AMP is below 1 mM. However, further increases in intracellular AMP did not generate a corresponding decrease in intracellular ATP levels. In addition, we observed that the intracellular ATP:ADP ratio was inversely linear (r^2^ = 0.977) to the concentration of extracellular AMP (Fig viii in S1 File). Since the cellular levels of ADP remained relatively constant, the large drop in ATP as a result of a smaller increase in AMP is rather perplexing. At a 1 mM extracellular AMP concentration, the erythrocytes’ intracellular ATP:AMP ratio dropped from over 100:1 to 25:1, a 4-fold change. Given that the intracellular ADP concentration appeared relatively constant, this suggests that either a significant amount of the ATP was rapidly degraded or lost from the erythrocytes.

### Influx of AMP induces ATP release from erythrocytes

**Fig 6:**
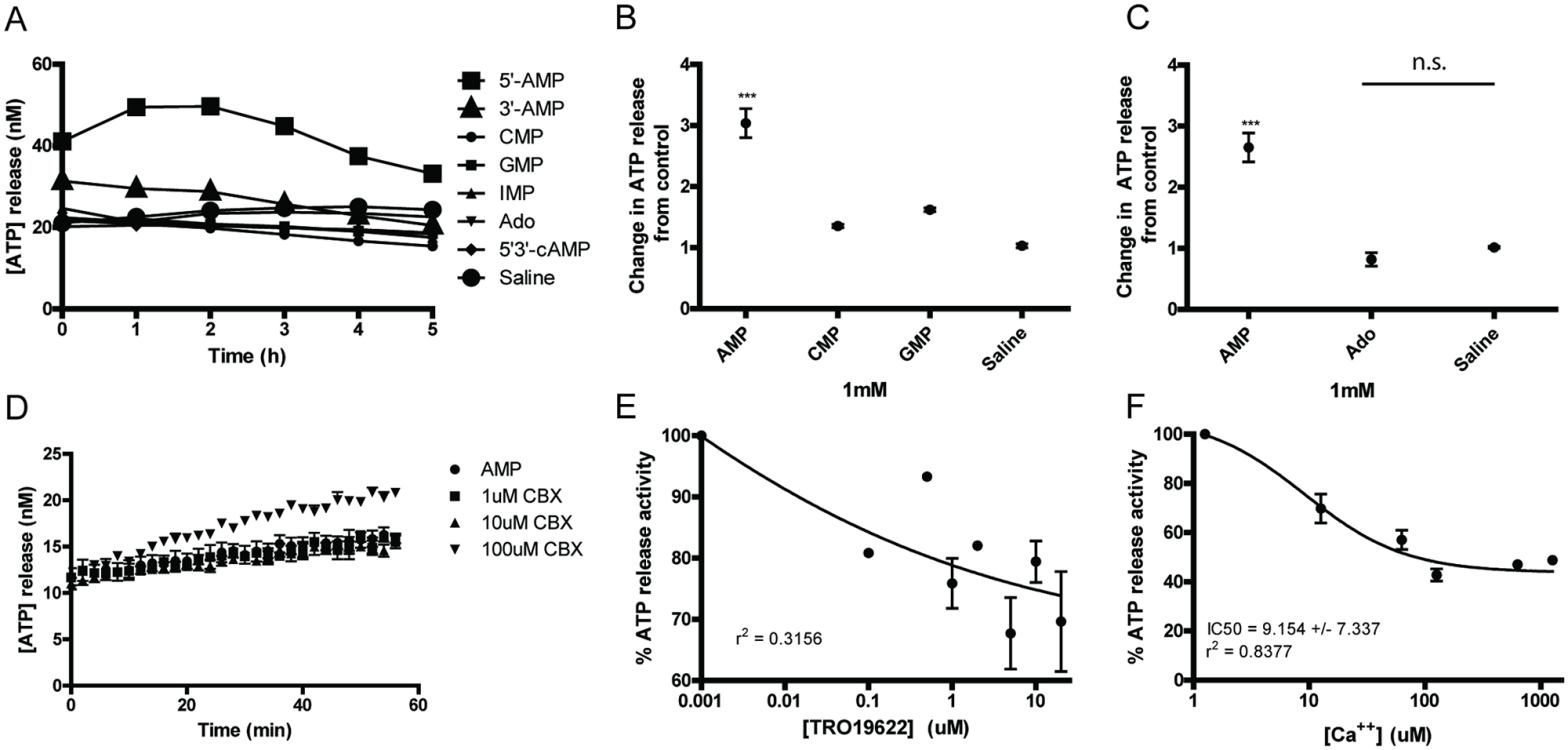
Nucleotides’ induction of ATP release by erythrocytes. (A) ATP release time course after addition of various nucleotides and adenosine measured by luciferase assays. (B) Quantification of multiple samples for ATP release stimulated by various nucleotides (N = 3). (C) Quantification of multiple samples of AMP and adenosine stimulated ATP release (N = 3). (D) Effect of CBX on ATP release stimulated by AMP. (E) Effect of TRO19622 on ATP release stimulated by AMP. (F) Effect of Ca^2+^ on ATP release stimulated by AMP. P values = *** < .0001.

The smaller than expected increase in intracellular ADP predicted by the adenylate equilibrium in response to an influx of AMP prompted us to investigate the possibility that intracellular ATP could have been released by the erythrocytes during the initial influx of AMP. Aware of the possibility that extracellular ATP can be quickly degraded by ATPase’s that are associated with erythrocytes’ membranes [1], a luminometer, (Lumicycle, Actimetrics, IL, USA), was used to monitor ATP release using an ATP-activated luciferase assay in real-time. Induced ATP release from isolated erythrocytes was measured when the erythrocytes were incubated with PBS (control), 5’-AMP, other nucleotides (3’-AMP, 5’-CMP, 5’-GMP, IMP, and 5’-3’-cAMP), or adenosine at 1 mM concentrations (Fig 6A). Interestingly, responses above the PBS control levels of ATP-activated luciferase activities were observed for 5’-AMP and to a small extent, 3’-AMP but not for the other nucleotides and nucleosides tested (Fig 6A and 6B). Again, adenosine, unlike 5’-AMP, was no more effective in inducing ATP release from erythrocytes than PBS (Fig 6C). Together, these studies demonstrate that influx of AMP into erythrocytes was accompanied by efflux of ATP, which could explain the absence of ADP increase predicted by adenylate equilibrium control.

Next, we investigated whether the efflux of ATP from erythrocytes that was stimulated by AMP influx could be inhibited by ion channel blockers. Studies have reported that ATP release by erythrocytes was mediated by the pannexin channel, PANX1, which is sensitive to blockage by carbenoxolone (10 μM) [22]. ATP release by erythrocytes induced by AMP was measured in the absence or presence of 1 μM, 10 μM and 100 μM carbenoxolone (Fig 6D). We observed that AMP-induced release of ATP from erythrocytes was not blocked by carbenoxolone. In fact, at the highest carbenoxolone concentration, we observed an additional AMP-mediated ATP release.

In another independent study, it was reported that the voltage-dependent anion channel (VDAC) inhibitor TRO19622 could inhibit ATP release from erythrocytes [23]. Therefore, we investigated whether AMP mediated ATP release by erythrocytes could be blocked by TRO19622. ATP released by erythrocytes stimulated by 1 mM AMP was titrated against increasing concentrations of TRO19622 (Fig 6E). We observed that erythrocyte ATP release stimulated by AMP was sensitive to inhibition by TRO19622 concentrations above 1 μM. These observations suggest that the channel that is mediating AMP influx and/or ATP efflux could be a member of the VDAC family. Since VDAC is known to be a major calcium exporter during apoptosis, we next investigated whether calcium could modulate AMP mediated ATP release from erythrocytes (Fig 6F). We observed a correlation between increased calcium concentrations up to 100 μM and inhibition of AMP-mediated ATP release from erythrocytes, with an IC_50_ (half maximal inhibitory concentration) value of ~9 μM. The observed maximum calcium inhibition is about 60% of the total ATP release. In addition to calcium, we investigated another divalent ion, magnesium. Unlike calcium, we observed that magnesium stimulated luciferase activity but had no effect on AMP-mediated ATP release by erythrocytes. These findings suggest that the AMP-mediated ATP release by erythrocytes is regulated by calcium. Together these studies demonstrate that the AMP mediated release of ATP by the erythrocytes is likely mediated by an ion channel/pore that is sensitive to inhibition by TRO196222 and calcium cations. Although inhibition of ATP release by tannic acid was observed, the direct inhibition of luciferase enzyme by tannic acid cannot be ruled out, thus rendering these findings ambiguous.

## Discussion

Our work established a reliable procedure for AIHM, during which the core body temperature of the animal eventually drops to about 1°C above ambient temperature typically for 4–9 h and the animal appears to enter a state resembling that of suspended animation. Investigators who study natural hibernation and torpor of mammals have come to the conclusion that AIHM is an inducible form of torpor [24]. At the gene expression level, we found that AIHM involves changes in the expressions of a relatively small number of genes. These changes are largely restored within 48 h post-induction of AIHM, providing molecular evidence that AIHM is a safe and reversible process. Interestingly, the circadian clock was found to be largely stalled at the gene expression level during AIHM, a feature also observed in natural hibernations [25]. Our metabolomics studies revealed that the 5’-AMP administered was largely catabolized by the time the animals entered AIHM and their urea cycle appeared to be functional, helping to avoid ammonia toxicity [26]. For potential applications of AIHM, one of our studies demonstrated that AIHM induces a reversible deep hypothermia that reduces ischemia/reperfusion damage following myocardial infarct [9]. Another study demonstrated that whole body cooling increased stabilization of a temperature-sensitive Cystic Fibrosis (CF) mutant protein, ΔF508-CFTR, improved its functions, alleviated CF pathological phenotypes and decreased mortality in CF mice [27]. Similar AIHM procedures are now used by independent research groups to cool experimental animals of various disease models [28][29][30][31][32].

The importance of CD73 and AMPD3 in modulating the response of the animal to AIHM was evident when comparing the responses from mice deficient in both CD73 and AMPD3 to those from wild type and single gene mutant mice. Mice with double deficiencies exhibited stronger responses to AIHM induced by either a sub-optimal or a normal dosage of AMP in comparison to wild type and the single gene mutant mice. Interestingly, loss of CD73 generated a stronger AIHM response than the loss of AMPD3, suggesting that extracellular catabolism of AMP by CD73 is a major regulatory step in preventing AIHM. The observations that the absence of AMPD3 further enhanced the AIHM response suggest that the level of the erythrocyte adenylate pool is fundamental to understanding the mechanistic process of AIHM. While our experiments further confirmed that AMP not adenosine regulates p50, the acute p50 changes in response to the same dose of AMP of erythrocytes from all four genotypes were indistinguishable.

While studying the stability and fate of extracellular AMP in whole blood of wild type and *Cd73*^*-/-*^ mice, we observed that AMP was rapidly taken up by both wild type and *Cd73*^*-/-*^ erythrocytes. Extracellular AMP was also rapidly catabolized by wild type but not *Cd73*^*-/-*^ whole blood, validating the conclusion that CD73 is the major extracellular catabolic enzyme for AMP [17]. In contrast to whole blood, incubation of AMP with isolated erythrocytes resulted in minimal degradation of AMP, indicating that CD73 is not associated with the outer membrane of erythrocytes. ADP likely does not enter the erythrocytes directly. The rapid conversion of ADP into AMP by isolated erythrocytes suggests that an ATPase is likely associated with the extracellular membrane, consistent with studies showing that a Mg^2+^ and Ca^2+^ dependent ATPase, CD 39, has been purified from erythrocyte membranes [1].

We found that erythrocyte uptake of AMP was unaffected by dipyridamole, thus AMP uptake was not the result of a conversion to adenosine entering the erythrocyte via ENT1. Our observation verified a previous report that AMP uptake by erythrocytes is unaffected by dipyridamole [6]. One study has suggested that isolated ANT was able to transport AMP [20]. Interestingly, an earlier proteomics study showed that ANT1 was expressed in reticulocytes, the immature erythrocyte [33], while another study suggested the expression of ANT in mature erythrocytes [34]. ANT is known to transport ATP out of the mitochondrial matrix in exchange for ADP from the intermembrane space. Carboxyatractyloside (CAT) was previously shown to inhibit ANT nucleotide transport in mitochondria [35]. We investigated whether a type of ANT was involved in AMP uptake by erythrocytes. We found that AMP uptake by the erythrocytes was not blocked by CAT, thus not mediated by a CAT-sensitive ANT. Tannic acid (TAN) is a large molecule inhibitor of various channels and transporters including the SLC25 transporter proteins [21]. Using a dose of 0.2% TAN, we observed a greatly reduced AMP uptake compared to controls. Even at 0.002% TAN, we observed a significant reduction in AMP uptake by erythrocytes. This inhibitory effect of TAN on AMP uptake suggests that the channel responsible for AMP uptake is either directly inhibited by TAN or that the presence of the large TAN complex nonspecifically blocks the channel. Given the broad range of inhibition by TAN of many channels and transporters, the identity of the AMP channel/transporter remains unclear.

In addition to pharmacological blockers, we further investigated whether there were natural nucleotides or metabolites that could modulate AMP uptake activity, presumably by competitive inhibition. We found that common metabolites such as glucose, lactate and urea did not inhibit AMP uptake by erythrocytes at similar concentrations to extracellular AMP. Nucleosides such as adenosine, also did not inhibit AMP uptake by erythrocytes indicating that the uptake of adenosine and AMP likely occurs through separate channels. These observations are consistent with the dipyridamole studies described earlier. We observed that the three adenine nucleotides and GMP were all able to reduce uptake of AMP by erythrocytes. However, a titration of AMP uptake against GMP as an antagonist demonstrated that AMP uptake was not significantly altered. The inhibition of AMP uptake by ADP and ATP is not surprising given that our studies and others have demonstrated the presence of an ATPase (CD39) associated with the membrane of erythrocytes [1]. It is possible that ATP and ADP are rapidly degraded to unlabeled AMP, which acts as a competitor to the radiolabeled AMP.

Studies have shown that erythrocytes export ATP in response to stress signals, one of which is low oxygen saturation, similar to conditions in our AIHM mice [36]. The above consideration led us to investigate whether erythrocytes’ ATP release can be triggered by uptake of AMP, using responses to other nucleotides and adenosine for comparison. Among the panel of nucleotides and nucleosides tested, including 5’-CMP, 5’-GMP, 5’-3’-cAMP, 5’-IMP, adenosine and 5’-AMP, only 5’-AMP consistently induced ATP release from erythrocytes. Addition of 3’-AMP also resulted in a release of ATP slightly greater than the PBS control.

We attempted to identify the transporter responsible for ATP release. A previous study reported that ATP release by erythrocytes could be blocked by a PANX1 specific inhibitor, carbenoxolone (CBX) [36]. We found that CBX was ineffective in inhibiting AMP induced ATP release by erythrocytes Thus, PANX1 is unlikely to be involved in ATP release induced by AMP uptake. Other studies have reported that the voltage dependent anion changer (VDAC) is known to exchange ATP for ADP in the inner mitochondrial membrane and it has been suggested a similar protein is located on the membrane of erythrocytes [23]. We found that TRO19622, a VDAC inhibitor, was effective in partially inhibiting the ATP release that was induced by extracellular AMP. VDAC is known to be inhibited by Ca^2+^, having divalent cation binding sites on the external interface of the protein [37]. We investigated whether divalent ions, namely Mg^2+^ and Ca^2+^, could inhibit ATP release. Mg^2+^ was unable to impact ATP release when added to the buffer, but Ca^2+^ was able to significantly inhibit ATP release. By measuring ATP release as a function of Ca^2+^ concentration, we found that maximal inhibition occurred around 100 μM Ca^2+^, with an IC_50_ of ~10 μM. These findings suggest that a VDAC type protein might be responsible for the AMP-induced ATP release, though both TRO19622 and Ca^++^ could only achieve partial inhibition.

Our studies further suggest that erythrocytes, which do not carry out *de novo* purine biosynthesis, regulate their intracellular adenine nucleotide levels via their ability to uptake AMP and release ATP. ATP release by erythrocytes into the extracellular matrix has been reported to be triggered by stress signals such as hypoxia and exercise [36]. Thus, erythrocytes, which utilize glycolysis as an ATP regenerator, are an ATP source for extracellular matrix functions, whereas mitochondria play a somewhat analogous role in the intracellular functions of nucleated cells. It has been shown that extracellular ATP has important biological functions, such as activating the P2Y family of receptors which help control vascular tissue relaxation and blood pressure [38]. We surmise that extracellular enzymes such as CD73 and ATPase/CD39 are part of the regulatory control of the entry of adenine nucleotides into erythrocytes. To release energy to drive various cellular activities, ATP and ADP undergo dephosphorylation by erythrocyte-associated ATPase to AMP. It is well known that extracellular AMP can also be dephosphorylated extracellularly by CD73 to adenosine that can activate adenosine receptor-mediated pathways, while excess adenosine is rapidly degraded by adenosine deaminase. Our study provides further evidence to show that extracellular AMP is readily taken up by erythrocytes, replenishing the erythrocyte adenine nucleotide pool, modulating hemoglobin oxygen affinity and thus tissue oxygen consumption.

## Supporting information

S1 File Supplementary figures. (DOCX)
