# Supplementary material for: New insights on the regulation of the adenine nucleotide pool of erythrocytes in mouse models
Source: PLoS One. 2017 Jul 26;12(7):e0180948. doi: 10.1371/journal.pone.0180948 (PMC5528878; doi:10.1371/journal.pone.0180948)
Supplement: S1 File — (DOCX) [file pone.0180948.s001.docx]

**Supporting Information: S1 File**

**(***New Insights on the Regulation of the Adenine Nucleotide Pool of Erythrocytes in Mouse Models* by William G. O’Brien III, Han Shawn Ling, Zhaoyang Zhao and Cheng Chi Lee**)**


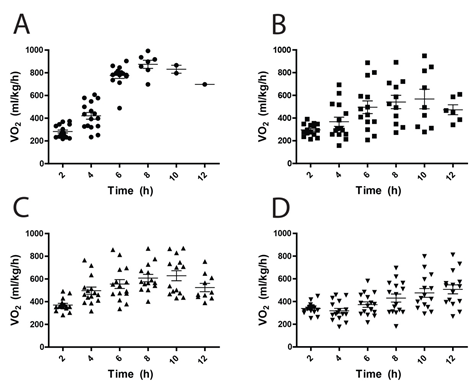


**Fig i: The time course of VO_2_ levels during AIHM of mice from each genotype.** A: wild type (N=16), B: *Ampd3^-/-^* (N=15), C:*Cd73^-/-^* (N=15), and D: *Ampd3^-/-^/Cd73^-/-^* (N=16)) were injected with the optimal dose of AMP (0.5 mg/gbw) and placed at an ambient temperature of 15^°^C. The length of time each mouse stayed in AIHM was calculated from injection to when their VO_2_ return to above 1200 mL/kg/h.

**Fig ii:**  **Average erythrocytes p50 values in vehicle and 2 mM Adenosine (N=3).**


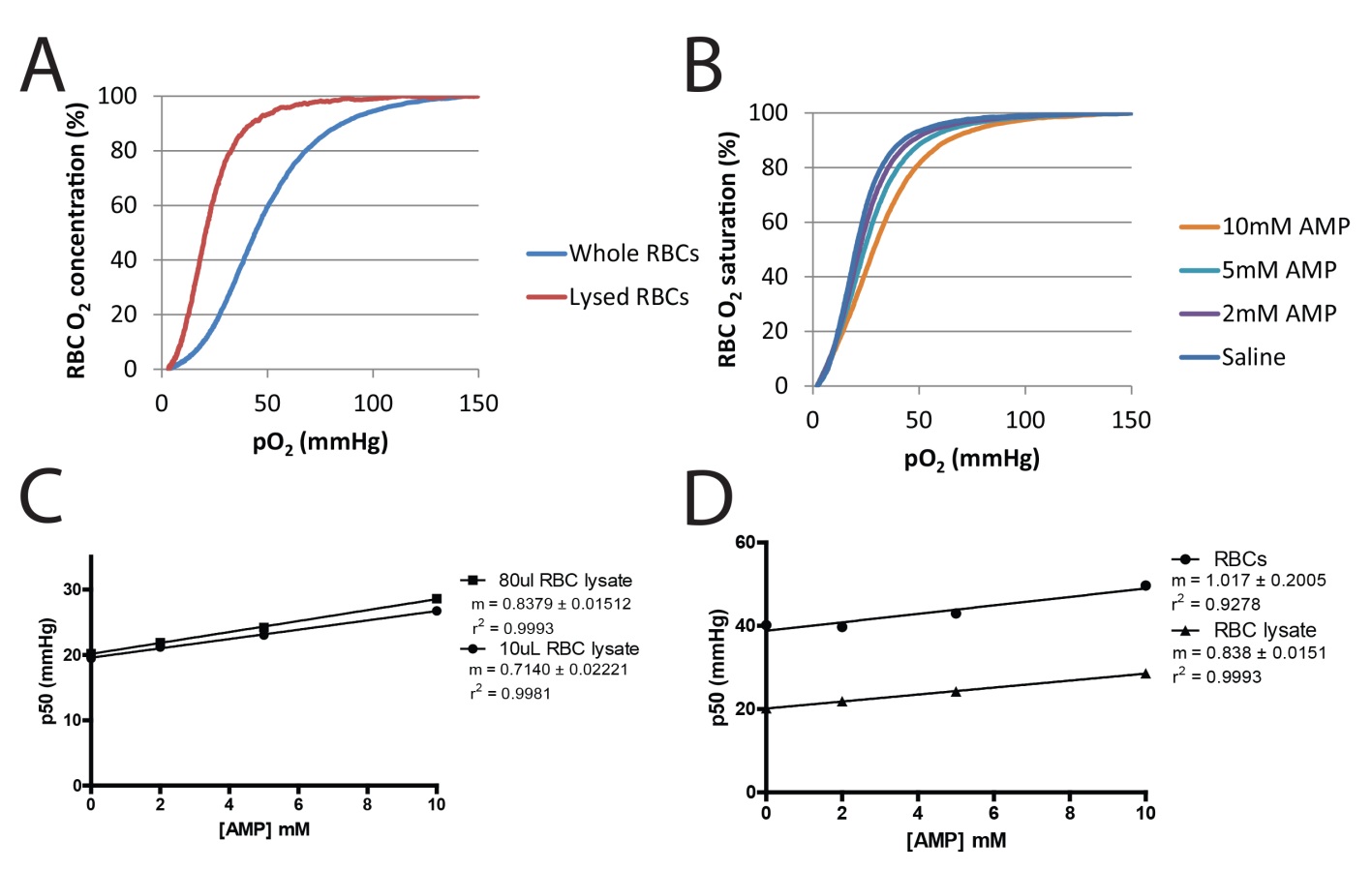


**Fig iii. Effect of AMP on lysed erythrocytes and intact erythrocytes.** A) Oxygen saturation curve of intact and lysed erythrocytes showing both a shift in baseline p50 value after lysis and a change in the curve shape. B) Oxygen saturation curve of erythrocyte lysate incubated with various concentrations of AMP. C) Plot of the change in p50 values in response to an AMP titration with different amounts of lysate. D) Plot of the change in p50 values in response to an AMP titration in intact and lysed erythrocytes.


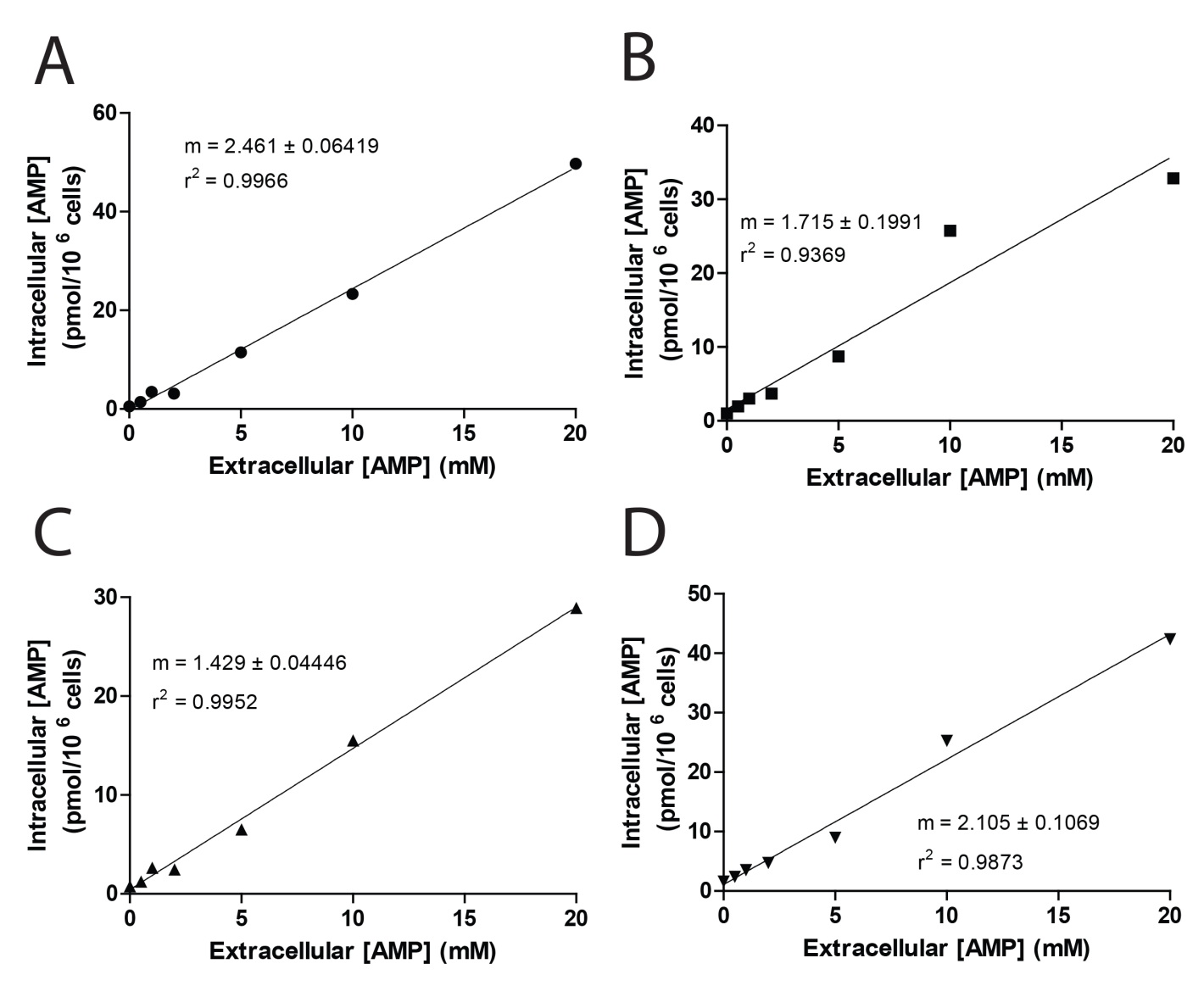


**Fig iv. Erythrocyte intracellular AMP after incubation with increasing concentrations of extracellular AMP** A-D) Erythrocytes intracellular levels of AMP in each of the four genotypes after extracellular AMP incubation (A: wild type, B: *Ampd3^-/-^*, C: *Cd73^-/-^*, and D: *Ampd3^-/-^/ Cd73^-/-^*).


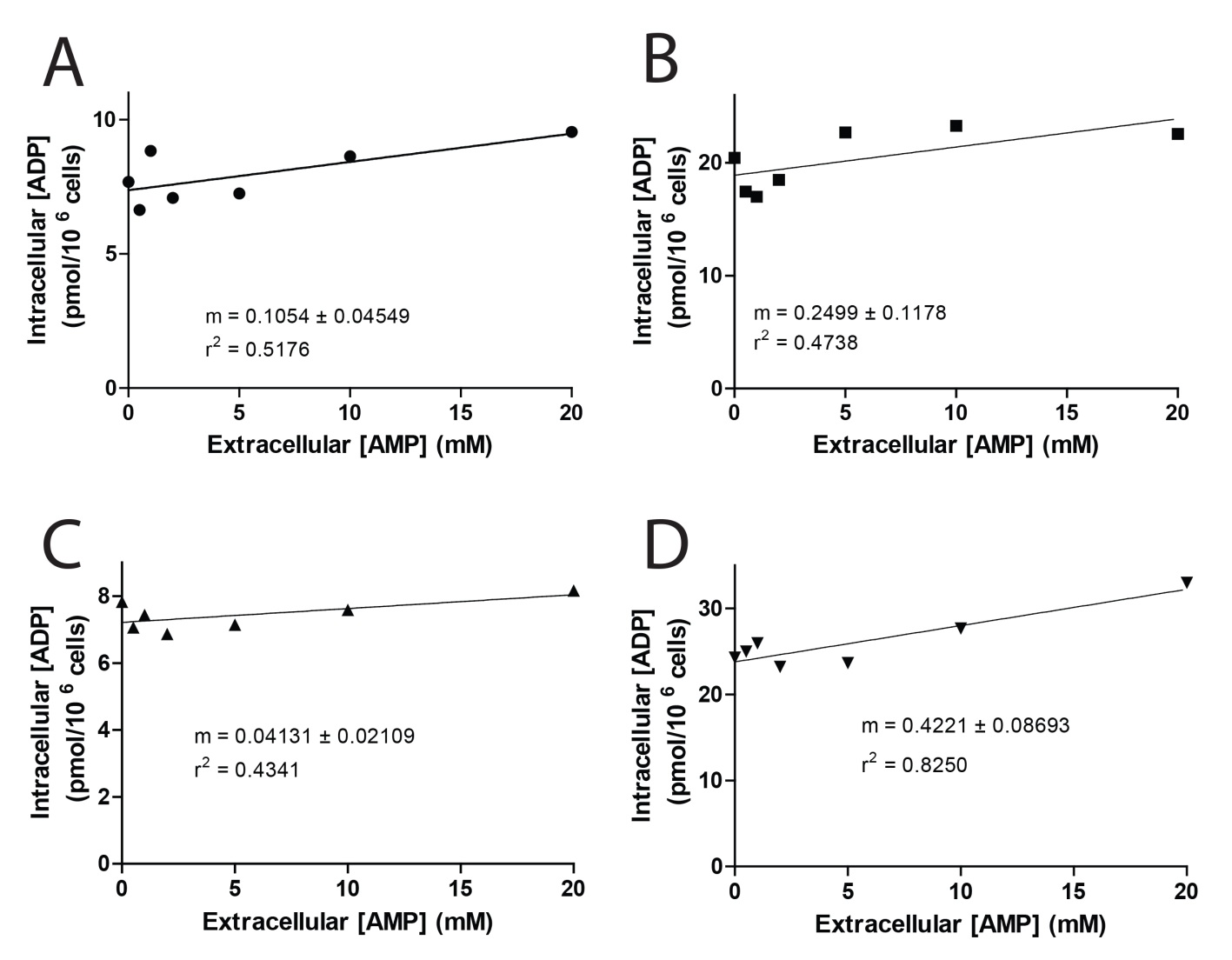


**Fig v. Erythrocyte intracellular ADP after incubation with increasing concentrations of extracellular AMP** A-D) Erythrocytes intracellular levels of ADP after extracellular AMP incubation in each of the four genotypes (A; wild type, B; *Ampd3^-/-^*, C; *Cd73^-/-^*, and D; *Ampd3^-/-^/ Cd73^-/-^*).


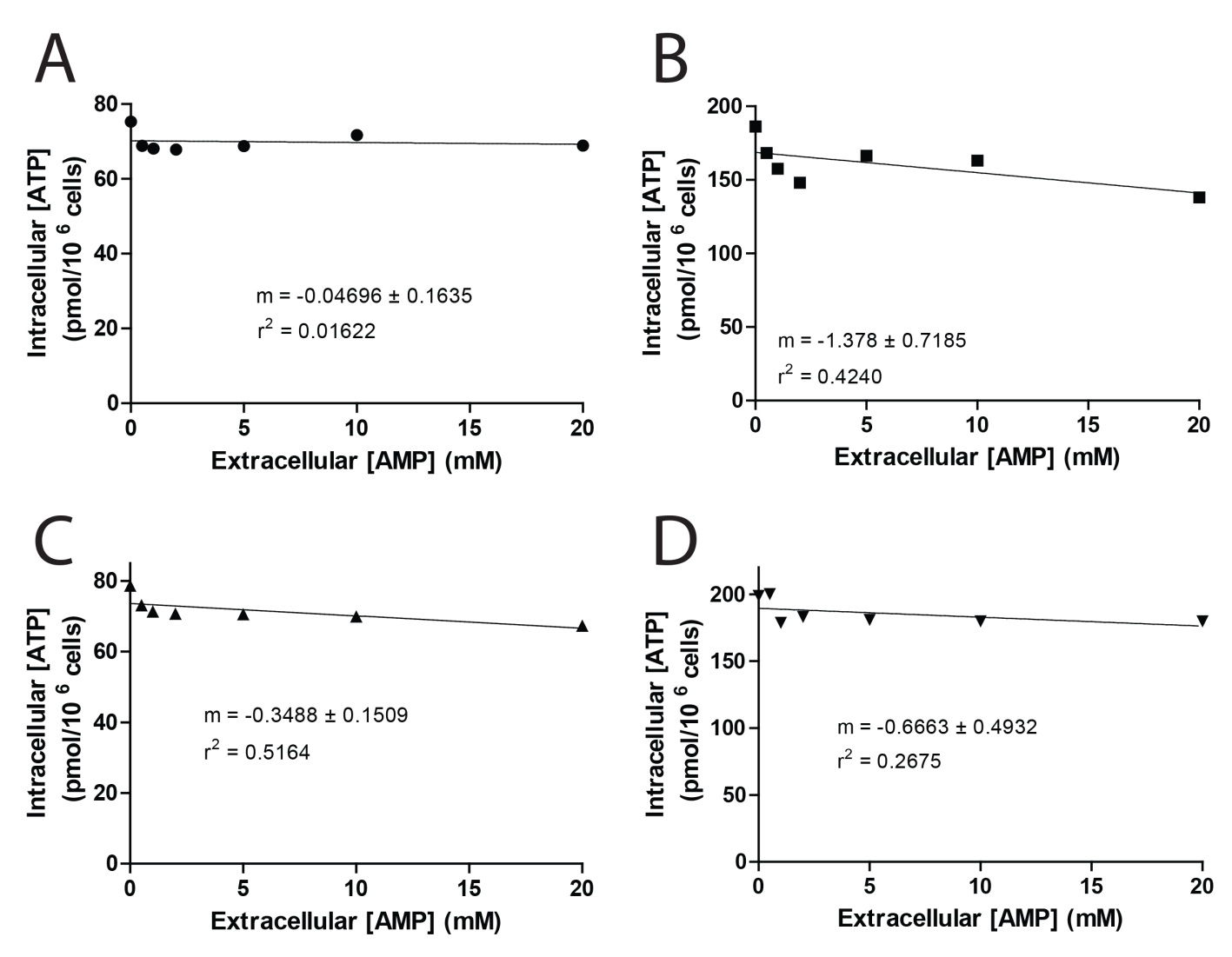


**Fig vi. Erythrocyte intracellular ATP after incubation with increasing concentrations** **of extracellular AMP** A-D) One plot for erythrocytes of each of the four genotypes (A; wild type, B; *Ampd3^-/-^*, C; *Cd73^-/-^*, and D; *Ampd3^-/-^/ Cd73^-/-^*).


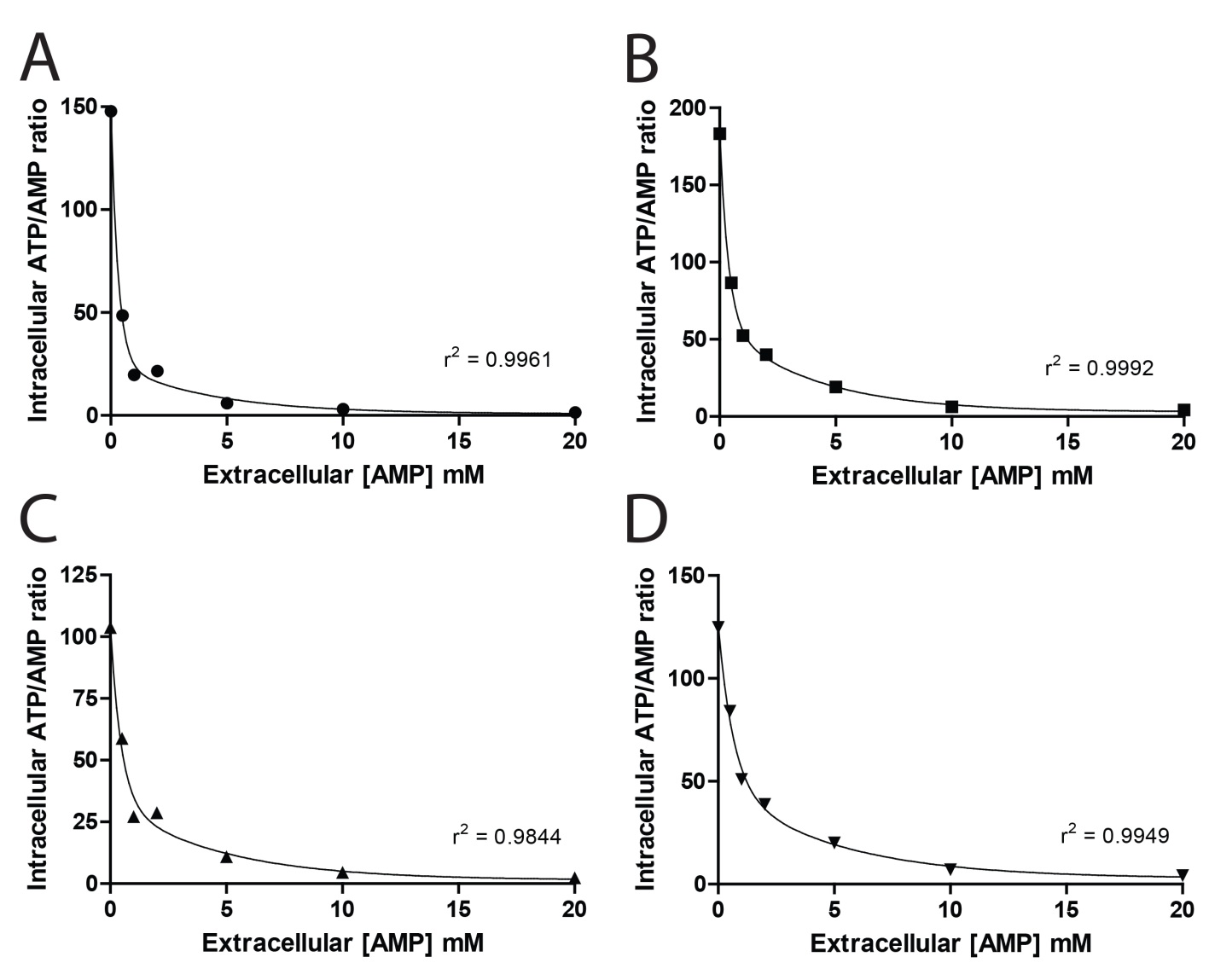


**Fig vii. Erythrocyte intracellular ATP:AMP ratio after incubation with increasing concentrations extracellular AMP.** A-D) One plot for erythrocytes of each of the four genotypes: (A; wild type, B; *Ampd3^-/-^*, C; *Cd73^-/-^*, and D; *Ampd3^-/-^/Cd73^-/-^*).


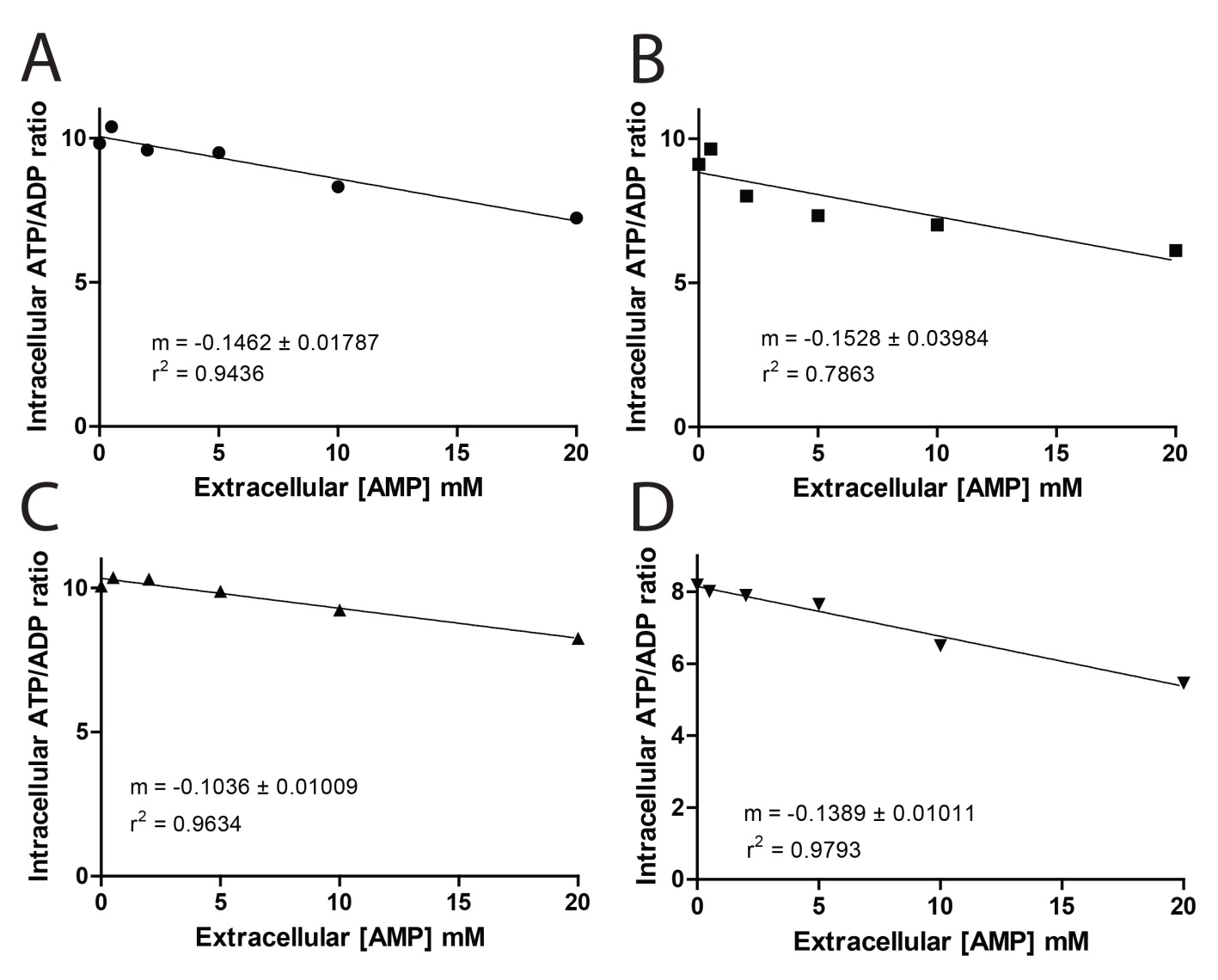


**Fig viii. Erythrocyte intracellular ATP:ADP ratio after incubation with increasing concentrations of extracellular AMP.** A-D) One plot for erythrocytes of each of the four genotypes (A; wild type, B; *Ampd3^-/-^*, C; *Cd73^-/-^*, and D; *Ampd3^-/-^/Cd73^-/-^*).
